# Demystifying the Relationship Between Metformin, AMPK, and Doxorubicin Cardiotoxicity

**DOI:** 10.3389/fcvm.2022.839644

**Published:** 2022-01-24

**Authors:** Manrose Singh, Akito T. Nicol, Jaclyn DelPozzo, Jia Wei, Mandeep Singh, Tony Nguyen, Satoru Kobayashi, Qiangrong Liang

**Affiliations:** ^1^Department of Biomedical Sciences, College of Osteopathic Medicine, New York Institute of Technology, Old Westbury, NY, United States; ^2^Department of Cardiology, The Second Affiliated Hospital of Xi'an Jiaotong University, Xian, China

**Keywords:** doxorubicin, metformin, AMPK, doxorubicin cardiotoxicity, cardio-oncology

## Abstract

Doxorubicin (DOX) is an extremely effective and wide-spectrum anticancer drug, but its long-term use can lead to heart failure, which presents a serious problem to millions of cancer survivors who have been treated with DOX. Thus, identifying agents that can reduce DOX cardiotoxicity and concurrently enhance its antitumor efficacy would be of great clinical value. In this respect, the classical antidiabetic drug metformin (MET) has stood out, appearing to have both antitumor and cardioprotective properties. MET is proposed to achieve these beneficial effects through the activation of AMP-activated protein kinase (AMPK), an essential regulator of mitochondrial homeostasis and energy metabolism. AMPK itself has been shown to protect the heart and modulate tumor growth under certain conditions. However, the role and mechanism of the hypothesized MET-AMPK axis in DOX cardiotoxicity and antitumor efficacy remain to be firmly established by *in vivo* studies using tumor-bearing animal models and large-scale prospective clinical trials. This review summarizes currently available literature for or against a role of AMPK in MET-mediated protection against DOX cardiotoxicity. It also highlights the emerging evidence suggesting distinct roles of the AMPK subunit isoforms in mediating the functions of unique AMPK holoenzymes composed of different combinations of isoforms. Moreover, the review provides a perspective regarding future studies that may help fully elucidate the relationship between MET, AMPK and DOX cardiotoxicity.

## Introduction

The anthracycline doxorubicin (DOX) has been widely used for over 5 decades and is a highly effective chemotherapeutic agent for the treatment of a broad spectrum of cancers including various solid tumors and leukemia. Unfortunately, DOX chemotherapy can cause severe cardiotoxic effects (1–3). Acute toxicity occurs immediately after treatment and is generally transient. Chronic cardiotoxicity is more serious and culminates in irreversible congestive heart failure. Currently, only the iron chelator dexrazoxane has been approved for limited clinical use for reducing DOX cardiotoxicity in certain pediatric or breast cancer patients (4–7). Given the continuing widespread use of DOX in cancer chemotherapies, it is imperative to identify new strategies that can protect against DOX cardiotoxicity without compromising the anti-tumor activity of DOX. Metformin (MET), a drug used for the first-line treatment of type 2 diabetes, has been suggested as such a dual-function agent that can simultaneously decrease DOX cardiotoxicity (8–11) and increase its anticancer activity (12, 13). The differential effects of MET on cardiomyocytes and cancer cells may be related to the differences in cellular energy metabolism. Cardiomyocytes are highly dependent on mitochondria for energy supply, while cancer cells primarily use glycolysis-generated ATP. Therefore, drugs such as MET that modulate mitochondrial function may have substantially different effects on the heart as compared to tumors. AMP-activated protein kinase (AMPK), a cellular energy sensor, is activated by MET and implicated in both cardioprotection and tumor growth. Most cell-based studies have suggested AMPK as a downstream effector of MET that functions to reduce DOX cardiotoxicity (9, 11, 14–17). However, the role of AMPK in cancer has been controversial (18). It remains uncertain whether and how AMPK affects the ability of MET to modulate DOX cardiotoxicity or tumor growth *in vivo*. This mini-review will extract evidence from currently available literature for or against a role of AMPK in MET-mediated protection against DOX cardiotoxicity. For the effects of MET and AMPK in antitumor therapies, the readers are referred to other review articles published elsewhere (18–24).

## Dox Cardiotoxicity is a Serious Clinical Problem

Dox is an extremely effective and wide-spectrum antineoplastic drug that can lead to dose-dependent cardiotoxicity, culminating in heart failure (1–3). This presents a serious problem to millions of cancer survivors who have been treated with DOX. Indeed, the cardiovascular mortality in cancer survivors exceeds that caused by cancer *per se* (25). DOX cardiotoxicity is even more significant in childhood cancer since about half of all pediatric patients are treated with anthracyclines and many childhood cancer survivors go on to develop cardiac dysfunction (26–28). Due to the dose-dependent risk, the lifetime cumulative dose of DOX has been recommended not to exceed 450 mg/m^2^ per patient (1). Thus, DOX cardiotoxicity is a significant life-long health concern for cancer survivors.

## Dox Induces Cardiotoxicity Via Multiple Mechanisms

**Figure 1:**
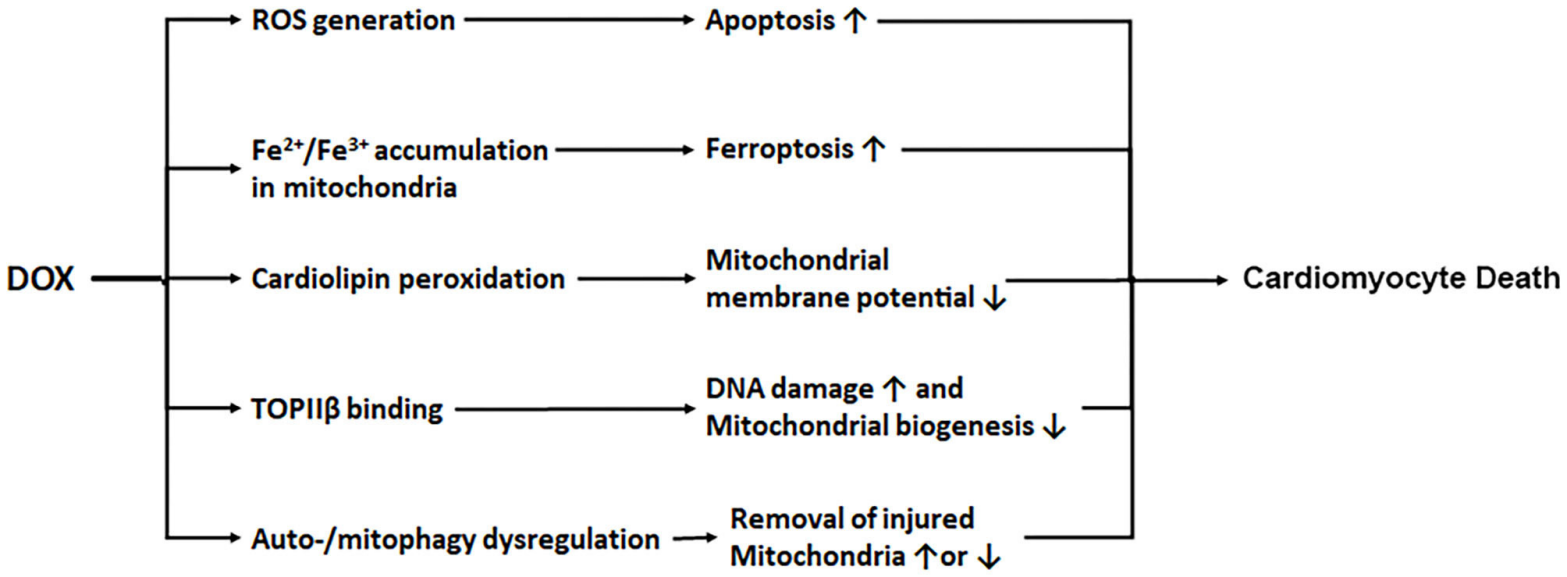
DOX induces cardiotoxicity via multiple mechanisms. DOX enters mitochondria triggering increased production of ROS, iron accumulation, cardiolipin peroxidation, and mitochondrial injury. DOX also binds to topoisomerase IIβ (TOPIIβ), resulting in DNA damage and reduced mitochondrial biogenesis. In addition, DOX causes autophagy/mitophagy dysfunction, leading to either reduced or excessive elimination of injured mitochondria, worsening cardiac injury.

Several mechanisms have been proposed to account for the ability of DOX to produce cardiotoxicity. DOX is concentrated in the mitochondria and its quinone moiety is reduced by the oxidoreductases to a semiquinone form which in turn donates its excess electron to O_2_, leading to the formation of reactive oxygen species (ROS) including superoxide anions (29, 30). Although the long-held ROS and oxidative stress theory of DOX cardiotoxicity is strongly supported by numerous animal studies (31–33), clinical trials have failed to demonstrate the efficacy of antioxidant supplements in reducing DOX-triggered cardiac injury (34, 35), suggesting that oxidative stress is not the only mechanism that mediates DOX cardiotoxicity. Interestingly, DOX has been shown to either bind with free iron (36) or cause mitochondrial iron accumulation in the heart (37), which may directly cause mitochondria-dependent ferroptosis or produce additional ROS intensifying the oxidative stress (38). The contribution of iron to DOX cardiotoxicity is demonstrated by the ability of the iron chelator dexrazoxane to attenuate DOX-induced cardiomyopathy (4, 5, 37). Another recognized culprit of DOX cardiotoxicity is mitochondrial dysfunction (39). Being the major site of DOX-induced ROS production, mitochondria themselves are vulnerable to oxidative injury. DOX interacts with the acidic lipoprotein cardiolipin in the inner mitochondrial membrane, resulting in its peroxidation and the opening of mitochondrial permeability transition pores which in turn triggers cytochrome c release and apoptosis (40, 41). The third mechanism proposed for DOX cardiotoxicity is through its effect on topoisomerase IIβ (TOPIIβ). While the antitumor effect of DOX is through DNA intercalation and TOPIIα inhibition (42–44), DOX also binds to TOPIIβ which is expressed mainly in quiescent cells such as cardiomyocytes. Mice null for TOPIIβ do not exhibit cardiotoxic effects with DOX treatment (45), suggesting that TOPIIβ is a major mediator of DOX cardiotoxicity. DOX is proposed to complex with TOPIIβ, leading to the activation of p53 mediated DNA damage pathways and the inhibition of genes implicated in mitochondrial biogenesis. Interestingly, dexrazoxane is shown to protect the heart by transiently depleting TOPIIβ levels in cardiomyocytes, suggesting that dexrazoxane may reduce DOX cardiotoxicity via both TOPIIβ depletion and iron chelation (46). The last potential mechanism of DOX cardiotoxicity relates to autophagy, a catabolic process for the cell to degrade long-lived proteins and organelles in the lysosome. The exact function of autophagy in DOX cardiotoxicity remains hotly debated, which is not surprising given the dynamic nature of the multi-step autophagic process and the numerous pathways implicated in its regulation. Indeed, DOX has been shown to either activate autophagy (17, 47–50) or inhibit autophagy (51–53), paradoxically, both of which contribute to cardiotoxicity. Adding to the confusion, DOX-triggered suppression of autophagy is seemingly cardioprotective (54). These conflicting results may be attributable to the differences in the experimental models used, the developmental stages of cardiomyopathy, and the dose and duration of DOX treatment, as well as the methods applied to manipulate different steps of the autophagic process and the techniques used to measure autophagic activities (49, 55). An early sign of DOX-induced mitochondrial damage is the loss of mitochondrial membrane potential (56–58). The latter is a major mechanism that triggers mitochondrial degradation by autophagy, a process known as mitophagy. However, as with autophagy, it remains controversial whether DOX activates or inhibits mitophagy and whether mitophagy contributes to or protects against DOX cardiotoxicity (59–62). Further investigation is needed to measure mitophagy flux and elucidate the role of mitophagy in DOX cardiotoxicity by using more reliable approaches and more clinically relevant animal models. In summary, it is likely that DOX induces cardiotoxicity via multiple mechanisms, including ROS generation, iron accumulation, cardiolipin peroxidation/mitochondrial injury, topoisomerase binding, and autophagy/mitophagy dysfunction (Figure 1).

## New Strategies to Diminish Dox Cardiotoxicity in Cancer Patients are Desperately Needed

The current approach for reducing DOX cardiotoxicity is to limit the overall cumulative dose of the drug. However, this also narrows the therapeutic window for cancer treatment. Other strategies for limiting its cardiotoxicity have been pursued. Attempts to develop chemical analogs that retain anti-tumor properties but have reduced cardiotoxicity have had minimal success (63). Liposomal DOX has improved pharmacokinetics and reduced accumulation in the heart (64) but has failed to replace conventional DOX for treatment of most solid tumors (65). An additional approach is to combine DOX with a cardioprotective agent during treatment. Common neurohormonal antagonists, such as β-adrenergic receptor blockers and angiotensin-converting enzyme inhibitors, are routinely used for treating non-cancer-related heart failure, but they are not recommended for preventing and managing DOX cardiotoxicity due to the marginal benefits and related adverse events (66). Currently, only the iron chelator dexrazoxane has been approved for clinical use for reducing DOX cardiotoxicity (4, 5). Unfortunately, dexrazoxane is not a ubiquitous treatment for anthracycline cardiotoxicity, and its use has been limited to pediatric patients with high risk acute lymphoblastic leukemia and breast cancer patients on high doses of DOX, given the possibility of dexrazoxane to cause myelosuppression and secondary malignancies (6, 67, 68). Therefore, it is imperative to develop new strategies to protecting against DOX-induced heart damage without compromising the anti-tumor activity of DOX. In this regard, the antidiabetic drug metformin (MET) has appeared to be such a promising dual-function agent that can improve the clinical use of DOX.

## Metformin Protects the Heart Against Various Pathological Conditions Including Dox Cardiotoxicity

**Figure 2:**
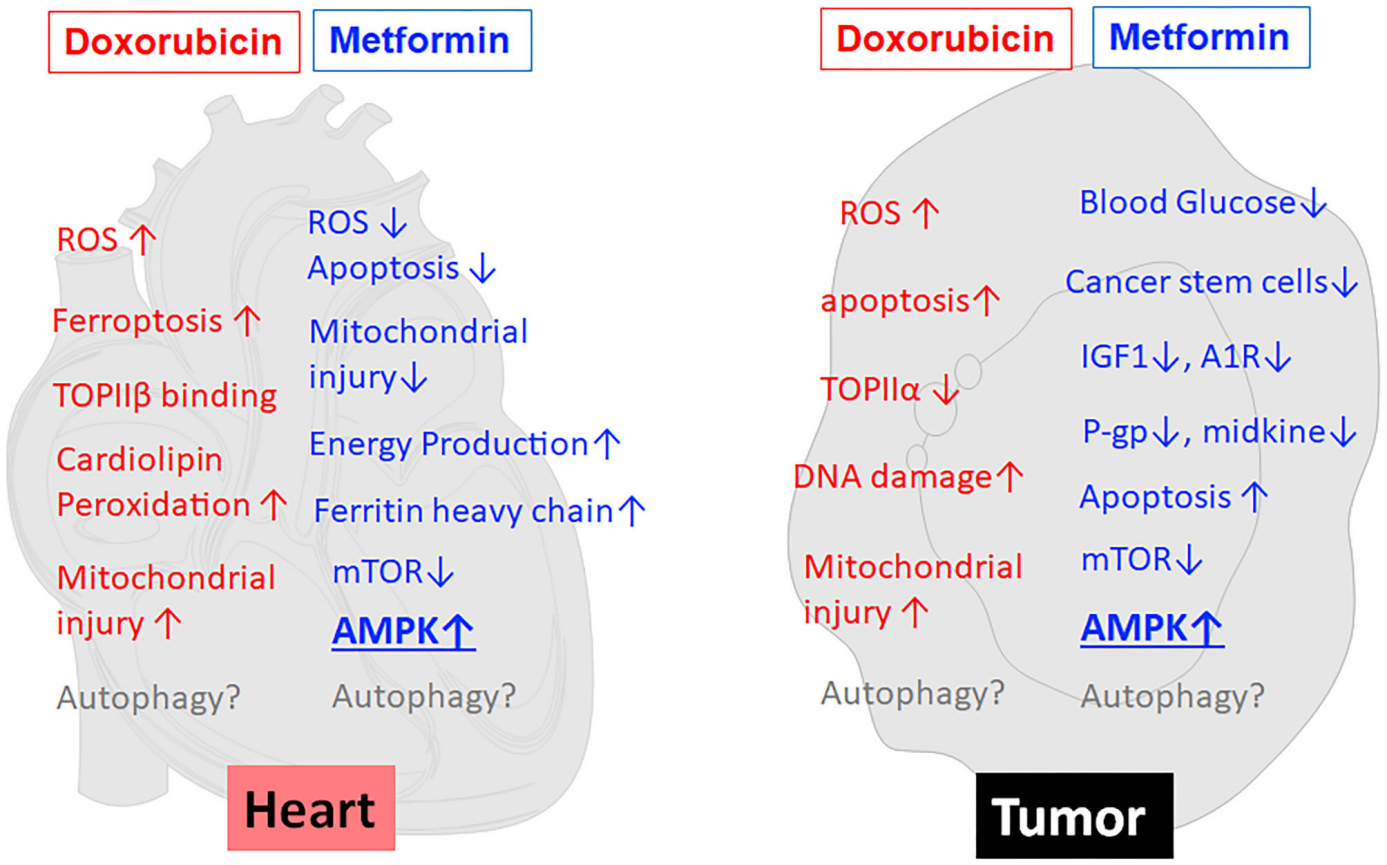
MET reduces the toxic effects of DOX on cardiomyocytes but concurrently enhances the anticancer effects of DOX on tumor cells. As shown in the left panel (heart), MET antagonizes DOX cardiotoxicity through several mechanisms, including attenuation of ROS generation and oxidative stress, inhibition of mitochondrial damage and maintenance of energy production, increased expression of ferritin heavy chain, and activation of AMPK. At the same time, MET enhances DOX antitumor effects (tumor, the right panel) through reduction of blood glucose, inhibition of cancer stem cells, reduction of IGF-1, modulation of adenosine A1 receptor (A1R), down-regulation of drug-resistant gene P-glycoprotein (P-gp), induction of apoptosis, inhibition of midkine, inhibition of mTOR, and activation of AMPK. Of note, AMPK activation has been suggested to be the major mechanism that mediates both the anti-tumor and cardioprotective effects of MET. On the other hand, the effects of MET on autophagy/mitophagy are not very clear. ↑, increase or upregulation; ↓, inhibition or downregulation; ROS, Reactive oxygen species; TOPII, Topoisomerase II; A1R, Adenosine A1 receptor; IGF1, Insulin-like growth factor 1; P-gp, P-glycoprotein.

Metformin (MET) is an oral biguanide agent that was first utilized to treat diabetes in France in 1957 (69) and approved by the US FDA in 1994 and has since been widely used as the first-line treatment for Type II diabetes due to its safety, efficacy and tolerability (70, 71). MET has been shown to protect the heart in people with or without diabetes mellitus (72). Indeed, MET is associated with decreased risk of heart failure (73) and reduced cardiovascular mortality independent of its glucose lowering effects (74). The cardioprotective effects of MET have been repeatedly confirmed by numerous pre-clinical studies under various cardiac conditions (75–79). Not surprisingly, MET can also reduce DOX cardiotoxicity in many animal studies (8–11). This may hold true in humans as well, given the ability of MET to attenuate radiation cardiotoxicity in breast cancer patients (80). Unfortunately, a phase II clinical trial “Use of Metformin to Reduce Cardiac Toxicity in Breast Cancer” was prematurely terminated due to its failure to meet target accrual (https://clinicaltrials.gov/ct2/show/NCT02472353). Apparently, further clinical trials are needed to confirm the cardioprotective effects of MET in cancer patients treated with DOX. MET has been suggested to antagonize DOX cardiotoxicity through several mechanisms (left panel in Figure 2), including attenuation of ROS generation and oxidative stress, inhibition of mitochondrial damage and maintenance of energy production (82), normalization of autophagy markers (8), increased expression of ferritin heavy chain in cardiomyocytes, and activation of AMP-activated protein kinase (AMPK) (11). The role of AMPK in MET-induced protection against DOX cardiotoxicity has been supported by numerous studies either in cultured cells or in animals (8–11).

## Metformin has Antitumor Properties that may Synergize With the Antitumor Activity of Dox

Several epidemiological studies, meta-analyses and animal studies have revealed that MET has anti-neoplastic and chemopreventive activities (20, 81) despite mixed results observed in other studies (82, 83). Indeed, diabetic patients taking MET have significantly reduced risk of cancer and lower cancer-related mortality (84–89). Several small-scale clinical trials have shown the ability of MET to induce favorable cellular and molecular changes in cancer patients (90–93). For example, clinical trials in pre-surgical endometrial cancer patients exhibited a significant decrease in Ki67 with MET monotherapy (19). Another study showed the ability of MET to inhibit the increase of Insulin-like growth factor 1 (IGF-1) and maintain the levels of IGF binding protein-1 although the progression-free survival was not affected (91). In addition, numerous animal studies have shown that MET can enhance the anticancer activity of DOX (11–13, 94, 95). Thus, it is highly desirable that large scale randomized clinical trials be conducted to confirm the usefulness of MET in cancer chemotherapy. Nevertheless, given the demonstrated anti-tumor and cardioprotective properties of MET, it is reasonable to believe that MET can be used in DOX-containing chemotherapy to enhance the antitumor activity of DOX and at the same time to reduce its cardiotoxic effect (96). Metformin is believed to exert its antitumor effects via multiple mechanisms (right panel in Figure 2), including activation of AMPK and inhibition of mTOR (13, 97, 98), reduction of blood glucose (21), reduction of insulin and IGF-1(98), inhibition of cancer stem cells (99), modulation of adenosine A1 receptor (100), down-regulating drug-resistant gene P-glycoprotein (P-gp) (94), inhibition of midkine (101), and induction of apoptosis (102, 103). Among them, AMPK activation has been suggested to be the major mechanism that mediates both the anti-tumor and cardioprotective effects of metformin (11, 13, 21, 97). If this is true, modulation of AMPK *per se* should improve the application of DOX in antitumor therapy.

## Ampk Signaling may Protect Against Dox Cardiotoxicity

AMP-activated protein kinase (AMPK) is a heterotrimeric protein kinase composed of a catalytic α subunit and two regulatory subunits (β and γ). Each subunit has multiple isoforms encoded by distinct genes (α1, α2, β1, β2, γ1, γ2, and γ3), and they combine to form 12 different AMPK holoenzymes (104). All isoforms except for γ3 are expressed in mouse and human heart, which can form 8 AMPK holoenzymes (105). As an energy sensor, AMPK detects and reacts to fluctuations in intracellular ATP levels under normal and stress conditions. The activated AMPK affects multiple metabolic pathways to maintain an energy homeostasis conducive to stress resistance and cell survival (106). There has been continuous intense research targeting AMPK for the treatment of multiple prevalent diseases, such as obesity, diabetes, cancer and cardiovascular diseases (107–109). Using AMPK deficient mice and chemical activators of AMPK such as AICAR and MET, numerous studies have shown that AMPK exerts a cardioprotective effect against myocardial ischemic injury (110, 111), diabetic cardiomyopathy (112), pathological cardiac remodeling (113), and heart failure (109). However, the use of MK-8722, a pan-AMPK activator, induces cardiac hypertrophy despite its ability to improve glucose homeostasis in rodents and rhesus monkeys (114), casting some doubt on the notion that AMPK activation always benefits the heart. Indeed, the gain-of-function mutations of the AMPK γ2 subunit result in severe cardiomyopathy in humans (115, 116), suggesting that the activation of some AMPK isoforms or holoenzymes can be detrimental to the heart under certain conditions. Interestingly, AMPK holoenzymes containing the α2 rather than the α1 subunit are the primary mediators of the cardiac phenotype of γ2 mutations (117), suggesting that α1-AMPK may play a different role than α2-AMPK, which underscores the complexity of isoform-specific functions of AMPK. This isoform-specific phenomenon was also observed in skeletal muscle where α2 but not α1 AMPK is responsible for AICAR-induced glucose uptake (118). When it comes to DOX cardiotoxicity, most cell-based studies have suggested AMPK as cardioprotective (9, 11, 14–17) despite the fact that DOX has been reported to either increase or decrease cardiac AMPK activity depending on the dose and duration of DOX treatment as well as the experimental models used (59, 119, 120). Pharmacological agents including MET, statins and many others can simultaneously activate AMPK and protect against DOX cardiotoxicity, but this remains an association and the causality between these two effects has not been established (119, 120). For example, the proposed role of AMPK in MET-mediated protection against DOX cardiotoxicity remains to be determined by using genetic animal models lacking AMPK function. Also, it remains essentially unknown which of the 8 isoform-specific AMPK holoenzymes mediates the putative protective effects on DOX cardiotoxicity *in vivo*.

## Ampk Plays Temporal and Isoform-Dependent Dichotomous Roles in Cancer

AMPK is considered to be both a tumor suppressor and an oncogene depending on the context (22). Studies have suggested AMPK as a tumor suppressor before disease arises, which is further enhanced by the biguanide phenformin. However, once cancer has occurred, AMPK becomes a tumor promoter to enhance cancer cell survival by protecting against metabolic, oxidative and genotoxic stresses (23). Indeed, the Liver Kinase B1 (LKB1)/AMPK pathway contributes to tumor cell survival by promoting cellular sensing of and adaptation to bioenergetic stress. Repression of LKB1 by miR-17~92 sensitizes MYC-dependent lymphoma to biguanide treatment (121). In addition, a loss of both AMPK α1 and α2 subunit isoforms in H-Ras-transformed mouse embryonic fibroblasts (MEFs) caused a complete failure of their growth *in vivo* in immunodeficient mice (122). However, a loss of AMPK α2 alone caused the tumors to grow more rapidly (123), suggesting isoform-dependent differential effects of AMPK on tumor growth. In summary, whether AMPK behaves as a tumor suppressor or a promoter depends on the developmental stage of the tumor and the specific isoform of the AMPK subunits.

## Met Activates Ampk, But it is Unknown if Ampk is Responsible for Cardioprotection By Met

Met has been shown to activate the AMPK pathway, and this has been proposed as the major mechanism that mediates the cardioprotective (9, 11, 109, 119, 120) and antitumor (13, 96, 97, 124) effects of MET. Thus, pharmacologically activating the AMPK pathway seems to be a two-birds-with-one-stone strategy to simultaneously reduce DOX cardiotoxicity and enhance its antitumor activity. However, it remains to be determined whether AMPK is indeed responsible for the potential double benefits of MET in humans or in clinically relevant animal models. Indeed, MET is shown to reduce pathological cardiac remodeling in the absence of AMPKα2 (76), suggesting the possibility that MET may reduce DOX cardiotoxicity independently of AMPK. Given the dual role of AMPK in tumor growth, it is equally unclear if the antitumor effects of MET are mediated by AMPK or its subunit isoforms.

## Summary and Future Perspectives

MET has been safely used to treat diabetes for several decades, making it a good candidate for repurposing (19). Indeed, many animal and preclinical studies suggest that MET has both cardioprotective and antitumor properties, which lends itself as a promising adjuvant drug for DOX anticancer therapies to reduce cardiotoxicity. MET is proposed to achieve these beneficial effects through the activation of AMPK that itself has been shown to protect the heart and modulate tumor growth under certain conditions. However, the role and mechanism of the hypothesized MET-AMPK axis in DOX cardiotoxicity and antitumor efficacy have not been firmly established. Convincing *in vivo* studies using tumor-bearing animal models and large-scale prospective clinical trials are needed to fully establish MET as an effective antitumor agent either alone or together with DOX. Also, the proposed role of AMPK in MET-mediated protection against DOX cardiotoxicity should be validated in genetic animal models lacking AMPK in the heart. Given the emerging evidence suggesting distinct functional roles of the AMPK isoforms, it is important to investigate how different AMPK holoenzymes containing unique combinations of isoforms will modulate the ability of DOX to affect either heart function or tumor growth. Future studies should also explore the cellular and molecular mechanisms that account for the differential responses of cardiomyocytes vs. cancer cells to DOX and MET, either individually or in combination. Without any doubt, answers to the above questions are expected to have a positive impact on the treatment of many types of cancers with DOX. For example, if it is firmly established that MET can reduce DOX cardiotoxicity and concurrently maintain its antitumor activity, the results could be rapidly translated into use for cancer patients because MET has been used in diabetic patients for decades. Specifically, including MET in a therapeutic protocol could reduce the amount of DOX needed to achieve the same antitumor effect. Alternatively, MET could make it possible to use larger doses of DOX to eradicate cancer more effectively without increasing cardiac damage. In short, MET could improve the therapeutic window for DOX, allowing greater flexibility in designing regimens for treating cancer. Finally, a comprehensive understanding of the relationship between DOX cardiotoxicity, antitumor efficacy, and individual isoforms of AMPK will guide novel mechanism-based therapeutic strategies that target AMPK.

## Author Contributions

ManrS, AN, JD, JW, MandS, and TN contributed to sections of the first draft. SK made the figures and edited the manuscript. QL wrote the outline, edited the draft, and finalized the manuscript. All authors contributed to the article and approved the submitted version.

## Funding

This line of research in the laboratory was supported by an in-house grant from New York Institute of Technology College of Osteopathic Medicine (NYITCOM) to QL and a research grant from the American Osteopathic Association (AOA, #2007816) to TN.

## Conflict of Interest

The authors declare that the research was conducted in the absence of any commercial or financial relationships that could be construed as a potential conflict of interest.

## Publisher's Note

All claims expressed in this article are solely those of the authors and do not necessarily represent those of their affiliated organizations, or those of the publisher, the editors and the reviewers. Any product that may be evaluated in this article, or claim that may be made by its manufacturer, is not guaranteed or endorsed by the publisher.

## Abbreviations

DOX: doxorubicin
MET: metformin
ROS: reactive oxygen species
AMPK: AMP-activated protein kinase
TOPIIα/β: topoisomerase IIα/β
ACE: angiotensin-converting enzyme
IGF1: Insulin-like growth factor 1
P-gp: P-glycoprotein
LKB1: Liver Kinase B1
MEFs: mouse embryonic fibroblasts.
